# Supplementary material for: sPLA2 Wobbles on the Lipid Bilayer between Three Positions, Each Involved in the Hydrolysis Process
Source: Toxins (Basel). 2022 Sep 26;14(10):669. doi: 10.3390/toxins14100669 (PMC9610741; doi:10.3390/toxins14100669)
Supplement: Supplementary file 1 [file toxins-14-00669-s001.zip › toxins-1908313-supplementary.pdf]

Supplementary Materials

sPLA2 Wobbles on the Lipid Bilayer between Three Positions, Each Involved in Hydrolysis Process

A

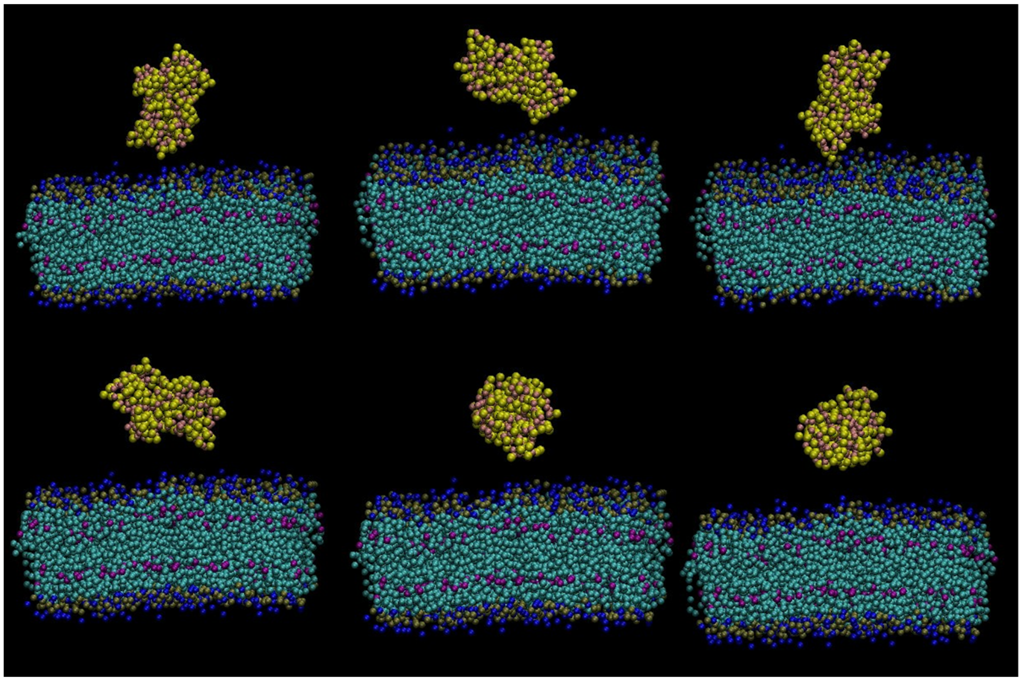

B

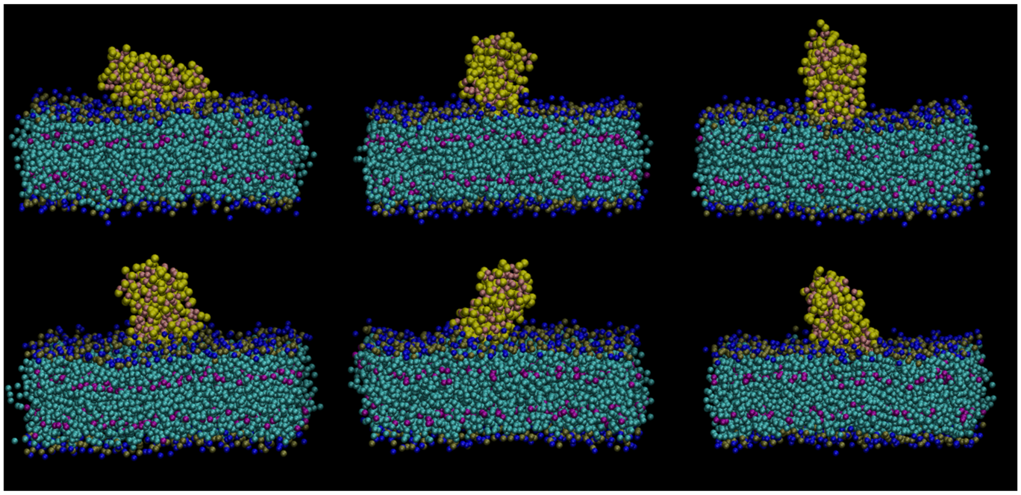

Figure S1. Coarse-grained dynamics for six different orientations of bvPLA2 relative to the bilayer **A.** Starting orientation of bvPLA2 above POPC bilayer in CG-MD. **B.** Final frame for each CG simulation after 5 μs.

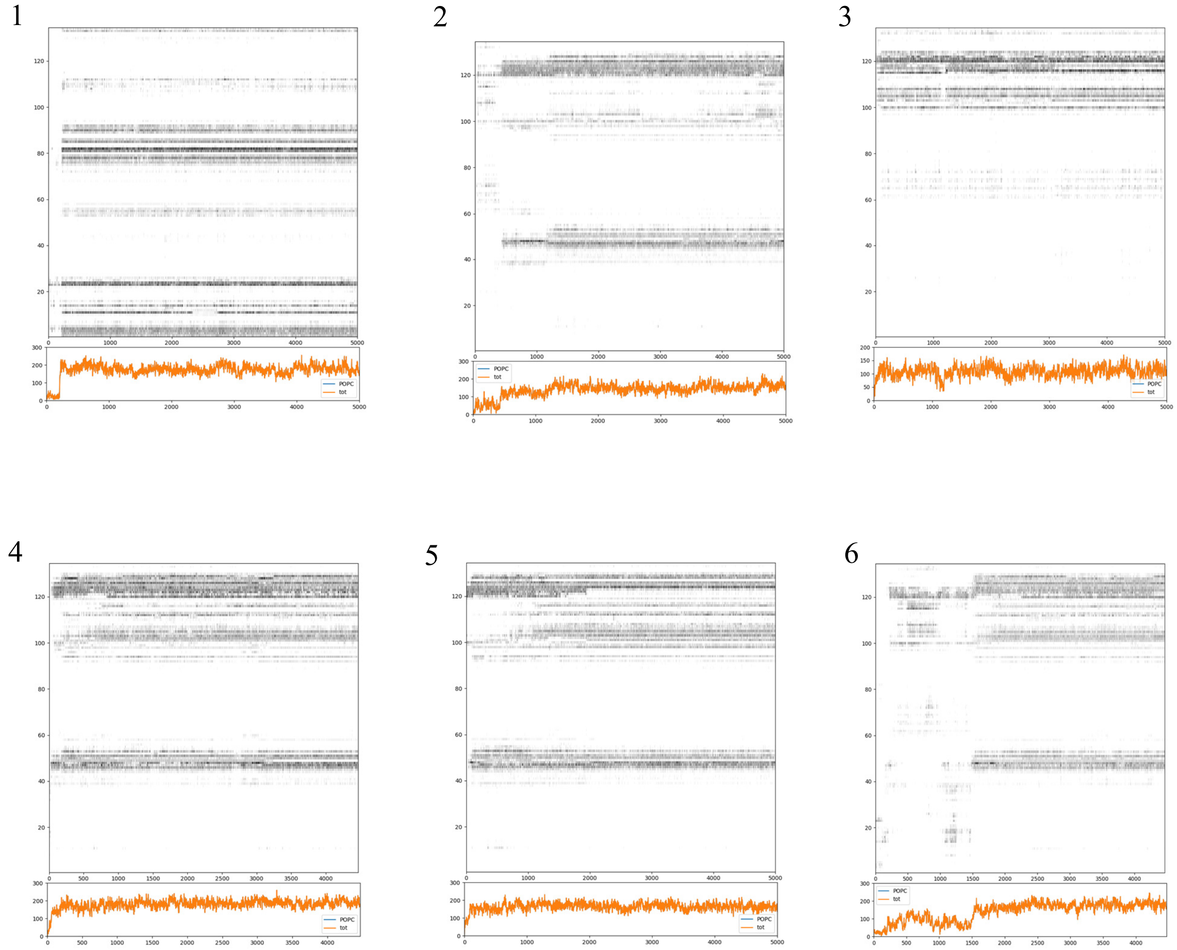

Figure S2. Coarse-grained dynamics analysis of contacts formation between bvPLA2 and all grains of POPC molecules for orientations 1–6 of bvPLA2 relative to the bilayer. X-axes represent simulation time, ns; Y-axes represent residue number; gray patterns correspond to contact formation (upper plots) and orange lines, total number of contacts (lower plots) per frame. Orientations 2, 4 ,5, and 6 were considered as the same pattern. Orientations 1, 2 and 3 were used for farther FA MD.

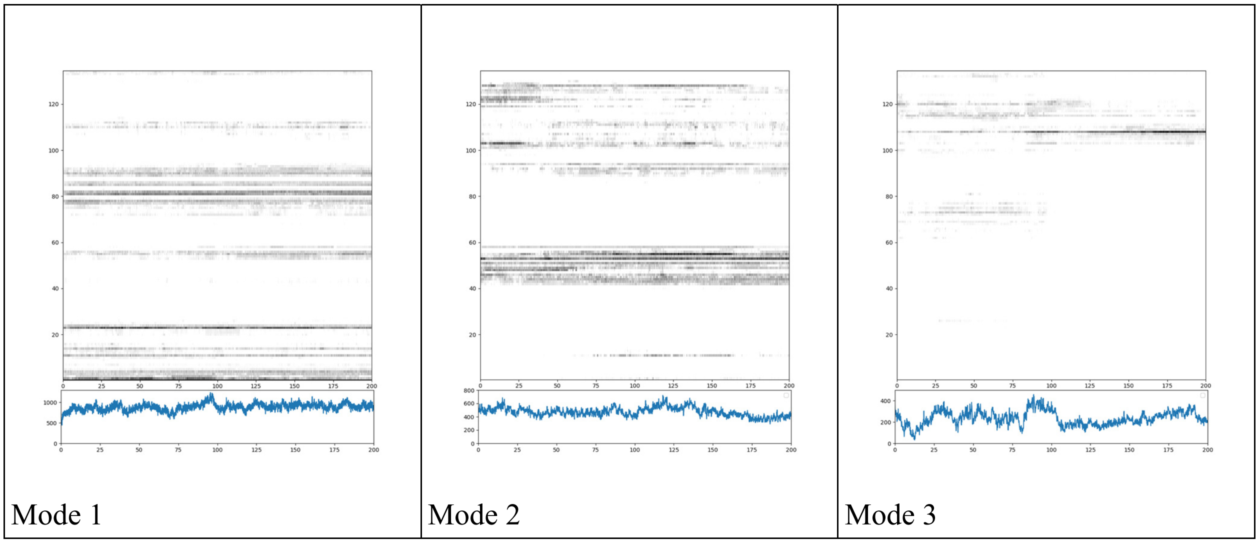

Figure S3. Full atom molecular dynamics analysis of contacts formation between bvPLA2 and all atoms of POPC molecules for three stable orientations of bvPLA2 relative to the bilayer. X-axes represent simulation time, ns; Y-axes represent residue number; gray patterns correspond to contact formation (upper plots) and orange lines, total number of contacts (lower plots) per frame.

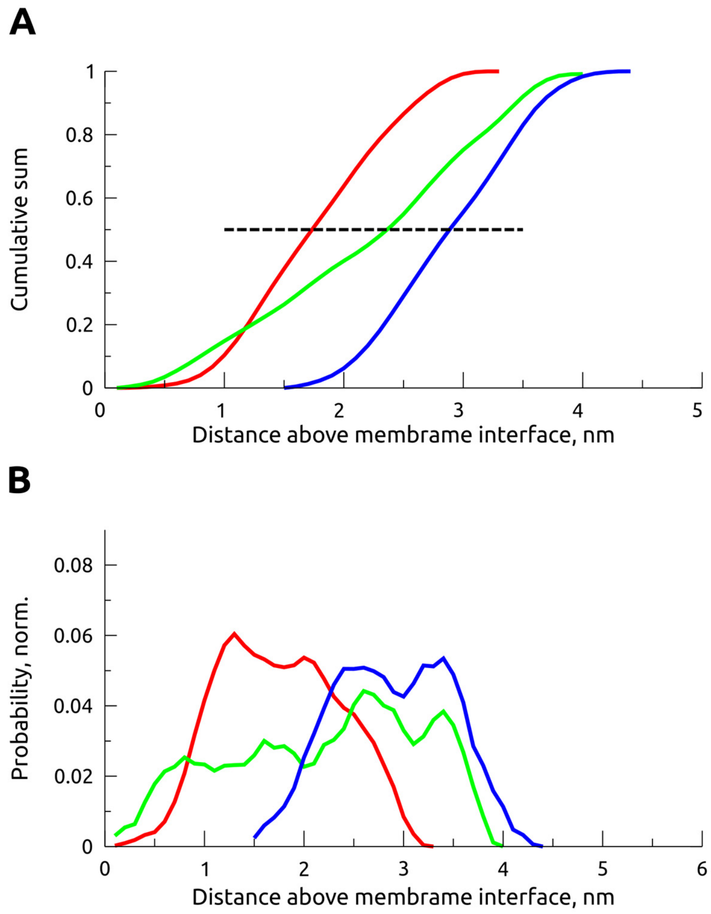

Figure S4. Analysis of protein altitude above membrane surface by full atom MD for three different modes, membrane interface was estimated as average position of the phosphorus atoms of the POPC molecule during simulation. Average altitudes were estimated to be 1.74 nm (mode 1, red line), 2.37 nm (mode 2, green line), and 2.90 nm (mode 3, blue line). **A**. Cumulative distribution of protein altitude for each mode, median value indicated by dash line. **B**. Probability distribution of protein altitude for each mode.

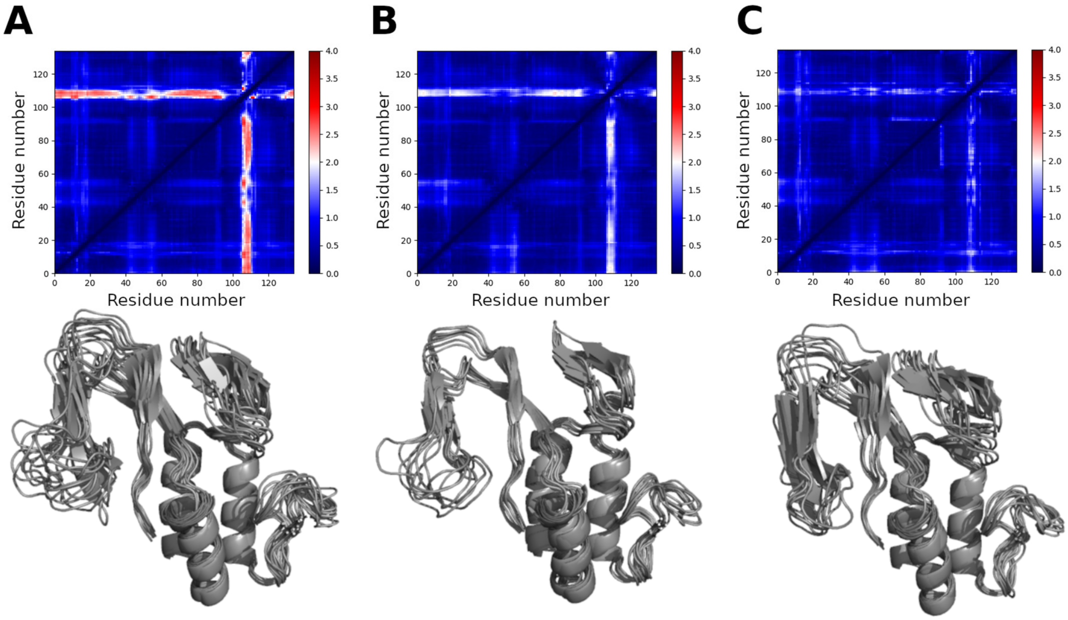

Figure S5. Cluster analysis (RMSD 2D plots of amino acids residues during FA-MD simulation) (**top**) and representative structures (**bottom**) of bvPLA2 in mode 1 (**A**), mode 2 (**B**) and mode 3 (**C**). Dark blue regions on diagram represent stable protein regions. Red regions represent highly mobile region of protein.

Table S1. Amino acids residues that form stable contact with membrane for each binding mode according to full atom molecular dynamic simulation.

Mode 1		Mode 2		Mode 3	
Residue	Contact intensity	Residue	Contact intensity	Residue	Contact intensity
Arg 23	88.2	Thr 53	51.1	Arg108	111.7
Phe 82	61.1	Ser 55	51.1	Glu107	21.3
Tyr 81	59.4	Thr 51	27.7	His115	13.5
Ile 1	48.6	Gly 44	26.7	Thr109	10.9
Pro 4	42.9	Trp 128	23.3	Glu106	10.2
Ser 55	41.3	Ala 43	21.5	Thr103	9.8
Leu 90	39.8	Ser 46	19.9	Glu110	7.7
Ile 78	37.3	Ala 54	18.5	Thr117	7.1
His 11	36.9	Glu 45	18.4	Cys105	6.3
Tyr 3	32.9	Asp 92	17.5	Ser121	5.5
Lys 85	29.5	Gly 49	16.6	Lys 120	5.2
Ile 91	26.1	Lys 94	15.0		
Met 86	24.0	Ser 42	14.6		
Phe 24	23.8	Arg58	14.5		
Thr 77	23.8	Thr 103	14.5		
Lys 14	23.6	His 11	13.1		
His 56	22.5	His 56	10.1		
Asp 92	21.5	Val 102	9.0		
Glu 110	21.2	Leu 50	8.3		
Ala 54	18.0	Gly 111	7.2		
Ile 2	16.3	Ile 91	6.7		
Asn 89	15.7	Arg 112	6.1		
Thr 53	16.6	Gln 127	5.8		
Arg 112	13.0	Leu 90	5.7		
Arg 58	12.8	Lys 122	5.2		
Ser 79	12.1				
Gly 5	10.3				
Ala 75	9.5				
Lys 72	8.7				
Thr 93	8.6				
Tyr 134	7.8				
Asp 76	7.5				
Val 83	6.9				
Gly 111	5.7				
